# Supplementary material for: Inhibition of PKCθ Improves Dystrophic Heart Phenotype and Function in a Novel Model of DMD Cardiomyopathy
Source: Int J Mol Sci. 2022 Feb 18;23(4):2256. doi: 10.3390/ijms23042256 (PMC8880527; doi:10.3390/ijms23042256)
Supplement: Supplementary file 1 [file ijms-23-02256-s001.zip › ijms-1563601-supplementary.pdf]

International Journal of
Molecular Sciences

MDPI

Supplementary materials

Inhibition of PKCθ Improves Dystrophic Heart Phenotype and Function in a Novel Model of DMD Cardiomyopathy

Jacopo Morroni [1], Leonardo Schirone [2], Valentina Valenti [3], Clemens Zwergel [4], Carles Sánchez Riera [1], Sergio Valente [4], Daniele Vecchio [2], Sonia Schiavon [2], Rino Ragno [4], Antonello Mai [4], Sebastiano Sciarretta [2,5], Biliana Lozanoska-Ochser [1,†] and Marina Bouchè [1,*,†]

[1] Department of Anatomical, Histological, Forensic Medicine and Orthopaedic Sciences, Section of Histology and Embryology, Sapienza University of Rome, Rome, 00161, Italy; jacopo.morroni@uniroma1.it (J.M.); carles.sanchezr@gmail.com (C.S.R.); biliana.lozanoska-ochser@uniroma1.it (B.L.-O.)
[2] Department of Medical and Surgical Sciences and Biotechnologies, Sapienza University of Rome, Rome, 00185, Italy; leonardo.schirone@uniroma1.it (L.S.); daniele.vecchio@uniroma1.it (D.V.); sonia.schiavon@uniroma1.it (S.S.); sebastiano.sciarretta@uniroma1.it (S.S.)
[3] Department of Cardiology, Ospedale Santa Maria Goretti, Latina, 04100, Italy; valevale2012@hotmail.com
[4] Department of Drug Chemistry and Technologies, Sapienza University of Rome, Rome, 00185, Italy; clemens.zwergel@uniroma1.it (C.Z.); sergio.valente@uniroma1.it (S.V.); rino.ragno@uniroma1.it (R.R.); antonello.mai@uniroma1.it (A.M.)
[5] Department of AngioCardioNeurology, IRCCS Neuromed, 86077 Pozzilli, Italy
* Correspondence: marina.bouche@uniroma1.it
† These authors contributed equally to this work.

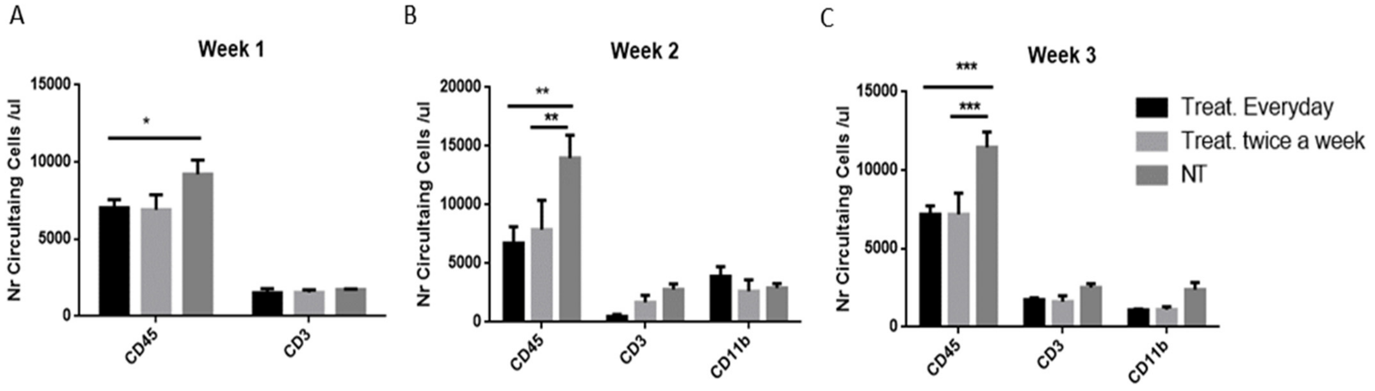

Figure S1. C20 twice administration is effective in reducing circulating CD3+and CD11b+ cells in mdx mice. Number of circulating CD3+and CD11b+ cells in mdx mice blood after 1 (**A**), 2 (**B**) or 3 (**C**) weeks of treatment (twice/week) by IP injection at the dose of 5 mg/kg. Data are presented as media of the CD3+ circulating number normalized per microliter of blood +/- SEM; n = 3 independent samples/group. * = $p < 0.05$, ** = $p < 0.01$, *** = $p < 0.001$, one-way ANOVA with Bonferroni correction for multiple comparisons.

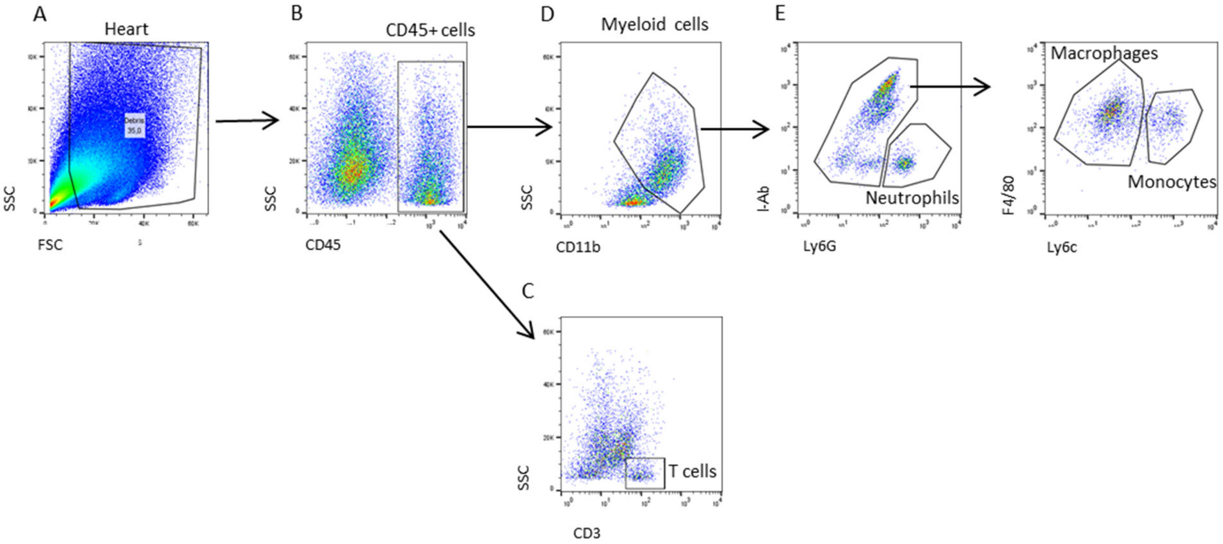

Figure S2. Cytofluorimetric gating strategy. (**A**) Heart infiltrating cells were isolated as described in Materials & Methods and analysed with the instrument CyAn ADP (DAKO) and FlowJo software 10.1. (**B**) Single, DAPI-negative (live) cells were then gated for their positivity to CD45 to identify the hematopoietic lineage. $CD45^+$ cells were then alternatively gated for their positivity to CD3 (T lymphocytes) (**C**) or CD11b (myeloid population) (**D**). Myeloid population was further divided into $Ly6g^+/I\text{-}Ab^-$ cells (neutrophils) and $Ly6g^-/I\text{-}Ab^+$ cells (**E**). $Ly6g^-/I\text{-}Ab^+$ population was then split in $F4/80^+/Ly6c^-$ cells (macrophages) and $F4/80^+/Ly6c^{hi}$ cells (freshly recruited monocytes) (**F**).

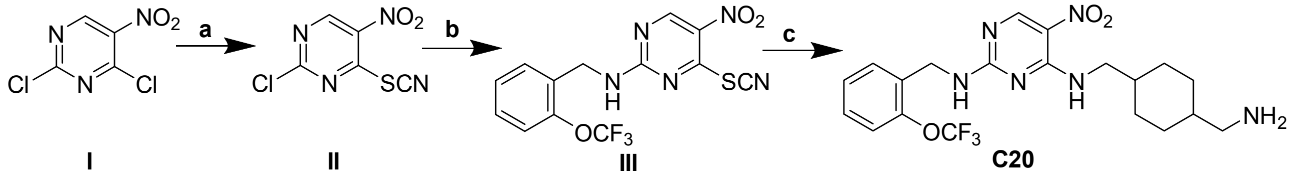

Figure S3. C20 synthesis. (**a**) KSCN, AcOH 0 °C. 2 h; (**b**) *o*-trifluoromethoxybenzylamine, TEA, abs EtOH, rt, 16 h; (**c**) 1,4-Cyclohexanebis(methylamine), DCM, rt, 16 h.
